# Comprehensive evaluation of Chinese peanut mini-mini core collection and QTL mapping for aflatoxin resistance

**DOI:** 10.1186/s12870-022-03582-0

**Published:** 2022-04-21

**Authors:** Yingbin Ding, Xike Qiu, Huaiyong Luo, Li Huang, Jianbin Guo, Bolun Yu, Hari Sudini, Manish Pandey, Yanping Kang, Nian Liu, Xiaojing Zhou, Weigang Chen, Yuning Chen, Xin Wang, Dongxin Huai, Liying Yan, Yong Lei, Huifang Jiang, Rajeev Varshney, Kede Liu, Boshou Liao

**Affiliations:** 1 Key Laboratory of Biology and Genetic Improvement of Oil Crops, Ministry of Agriculture and Rural Affairs, Oil Crops Research Institute of Chinese Academy of Agricultural Sciences (OCRI-CAAS), Wuhan, 430062 China; 2 National Key Laboratory of Crop Improvement, Huazhong Agricultural University, Wuhan, 430070 China; 3 International Crops Research Institute for the Semi-Arid Tropics (ICRISAT), Hyderabad, 502324 India

**Keywords:** *Aspergillus flavus*, Peanut, Shell infection resistance, Seed infection resistance, Aflatoxin production resistance, Genome-wide association study (GWAS)

## Abstract

**Background:**

Aflatoxin contamination caused by *Aspergillus* fungi has been a serious factor affecting food safety of peanut (*Arachis hypogaea* L.) because aflatoxins are highly harmful for human and animal health. As three mechanisms of resistance to aflatoxin in peanut including shell infection resistance, seed infection resistance and aflatoxin production resistance exist among naturally evolved germplasm stocks, it is highly crucial to pyramid these three resistances for promoting peanut industry development and protecting consumers’ health. However, less research effort has been made yet to investigate the differentiation and genetic relationship among the three resistances in diversified peanut germplasm collections.

**Results:**

In this study, the Chinese peanut mini-mini core collection selected from a large basic collection was systematically evaluated for the three resistances against *A. flavus* for the first time. The research revealed a wide variation among the diversified peanut accessions for all the three resistances. Totally, 14 resistant accessions were identified, including three with shell infection resistance, seven with seed infection resistance and five with aflatoxin production resistance. A special accession, Zh.h1312, was identified with both seed infection and aflatoxin production resistance. Among the five botanic types of *A. hypogaea*, the *var. vulgaris* (Spanish type) belonging to subspecies *fastigiata* is the only one which possessed all the three resistances. There was no close correlation between shell infection resistance and other two resistances, while there was a significant positive correlation between seed infection and toxin production resistance. All the three resistances had a significant negative correlation with pod or seed size. A total of 16 SNPs/InDels associated with the three resistances were identified through genome-wide association study (GWAS). Through comparative analysis, Zh.h1312 with seed infection resistance and aflatoxin production resistance was also revealed to possess all the resistance alleles of associated loci for seed infection index and aflatoxin content.

**Conclusions:**

This study provided the first comprehensive understanding of differentiation of aflatoxin resistance in diversified peanut germplasm collection, and would further contribute to the genetic enhancement for resistance to aflatoxin contamination.

**Supplementary Information:**

The online version contains supplementary material available at 10.1186/s12870-022-03582-0.

## Background

Peanut or groundnut (*Arachis hypogea* L*.*) is an important oilseed and cash crop grown in more than 100 countries worldwide, with China, India and the USA being the leading producers. From 2009 to 2019, the global peanut production increased from 37.36 million tons to 48.76 million tons (FAO, 2020), which has greatly contributed to food supply and rural development in many Asian and African countries. Peanut not only contains nutritious oil, protein, sugar, vitamins and minerals for human consumption, but also plays an important role in sustainable agriculture with its wide adaptation to marginal soils, drought tolerance and capacity of fixing nitrogen. However, peanut is among the crops that could be easily contaminated by aflatoxins in both pre-harvest and post-harvest stages [1, 2]. Integrated management approaches for reducing risk of aflatoxin contamination in peanut are highly crucial in most regions in the world.

Aflatoxins, a type of polyketide-derived secondary metabolites produced by several *Aspergillus* fungi including *A. flavus and A. parasiticus*, are highly toxic and carcinogenic to humans and animals and hard to be eliminated from contaminated materials [3]. Aflatoxin contamination is an important factor affecting food safety of several sensitive crops including peanut. Peanuts could be infected by *A. flavus* and *A. parasiticus* in pre-harvest, during harvest and post-harvest stages [4]. To prevent and control aflatoxin contamination in peanut, several control strategies including applying biological agents, planting resistant peanut cultivars, implementing essential irrigation at later growth stage, controlling storage and transportation conditions have been extensively used [5, 6], among which, utilization of genetic resistance to aflatoxin in peanut varieties has been regarded as a core strategy.

Mixon and Rogers [7] were the first to suggest using resistant peanut cultivars to control aflatoxin contamination. Many peanut germplasm accessions with resistance to *A. flavus* infection and toxin production were identified in the past five decades [8–11]. In general, the resistance to aflatoxin contamination in peanut consists of three components or mechanisms including:a) shell infection resistance, b) seed infection resistance, and c) aflatoxin production resistance [12]. Waliyar et al. [9] identified three peanut cultivars (55–437, J11 and PI337394 F) and an ICRISAT breeding line (ICGV87710) with stable resistance to seed infection or aflatoxin production from testing 25 lines including cultivars grown in multiple environments in West Africa. Holbrook [10] found that the drought-tolerant peanut genotypes PI145681 and Tifton 8 had resistance to aflatoxin. Lei et al. [13] found that two genotypes, Thaishan Zhenzhu and Zhonghua 6 with resistance to bacterial wilt, possessed resistance to aflatoxin production. Jiang et al. [14] tested 561 accessions of Chinese peanut core collection and 155 accessions of ICRISAT peanut mini core collection and identified 8 genotypes with resistance to *A. flavus* infection or aflatoxin production. Dieme [11] tested 67 peanut genotypes under laboratory conditions and identified a resistant genotype 12CS-104 with an aflatoxin contamination level lower than the European Union standards (4 ppb). Compared to undamaged pods, damaged peanut pods had higher aflatoxin content [15], indicating that the pod shell might act as the preliminary physical protection mechanism against fungal infection. Qiu et al. [16] tested the shell infection resistance in 276 accessions and identified two resistant lines with low shell infection level. Relatively, less research efforts have been made for systematic evaluation and resistance discovery for shell infection resistance in diversified peanut germplasm collections.

Several studies have been carried out for inheritance of resistance to *A. flavus* infection and toxin production in peanut. Liang et al. [17] reported identification of six quantitative trait loci (QTLs) associated with *A. flavus* infection using SSR-based genetic linkage map. Yu et al. [18] assessed the seed infection indexes (SDIIs) and contents of aflatoxin AFB_1_ and AFB_2_ in kernels of a recombinant inbred line (RIL) population (developed from a cross of Zhonghua 10 (susceptible) × ICG 12625 (resistant)) harvested from three environments, and identified two QTLs for SDII while 12 QTLs for aflatoxin production. Based on a RIL population derived from a cross between Xinhuixiaoli (resistant) and Yueyou 92 (susceptible), Khan et al. [19] identified two QTLs related to resistance on chromosomes A03 and B04, respectively. Genome-wide association study (GWAS) has been employed to identify associated loci and candidate genes for resistance to *A. flavus* in maize and peanut. Warburton et al. [20] reported the results of GWAS of 107 SNPs associated with aflatoxin accumulation in one or more environments using 300 test-crossed maize hybrid lines. Zhang et al. [21] identified a major QTL for aflatoxin resistance by both QTL mapping of 228 RILs and GWAS of 437 maize inbred lines. Han [22] performed a GWAS of 313 maize inbred lines which were inoculated with *A. flavus* and identified four associated loci and 16 candidate genes. In peanut, Yu et al. [23] identified 60 associated SNPs through GWAS in the Chinese peanut mini-mini core collection. It is worth to mention that the identified resistant loci for SDII and aflatoxin accumulation were different, indicating the potential of pyramiding these two different resistances by marker-assisted breeding.

It has been proven that genetic enhancement for aflatoxin resistance in peanut is difficult even though considerable progress has been achieved in germplasm discovery and breeding. As any of the available three resistance mechanisms could not well resolve the contamination problem individually, it is highly necessary to pyramid the three resistances into one genetic background [24]. However, limited research has been conducted for comprehensive evaluation on all the three resistances in diversified peanut germplasm collections. In order to characterize and utilize germplasm resources more efficiently for peanut genetic improvement, selecting mini core based on the entire basic collection has been in many crops including peanut worldwide. For the Chinese peanut germplasm, a mini-mini core collection consisting of 99 accessions was selected from more than 6500 basic accessions including the landraces, improved varieties and introduced materials from foreign countries, and the mini-mini core well cover the entire variation of 21 phenotypic characters in the basic collection. The 99 accessions were systematically evaluated for their three resistances including that to shell infection, seed infection and toxin production in kernels in this research. Several elite germplasm lines with shell and seed infection resistance, and aflatoxin production resistance were identified. Using the RAD-Seq based genotyping data, GWAS was employed to identify SNP markers and candidate genes associated with shell infection, seed infection and toxin production resistances. The interesting results of this study would provide theoretical and germplasm basis for synergistic improvement of aflatoxin resistance by pyramiding different resistance mechanisms into high yielding genetic background.

## Results

### Variation of three resistances to *A. flavus* in the Chinese peanut mini-mini core

**Fig. 1:**
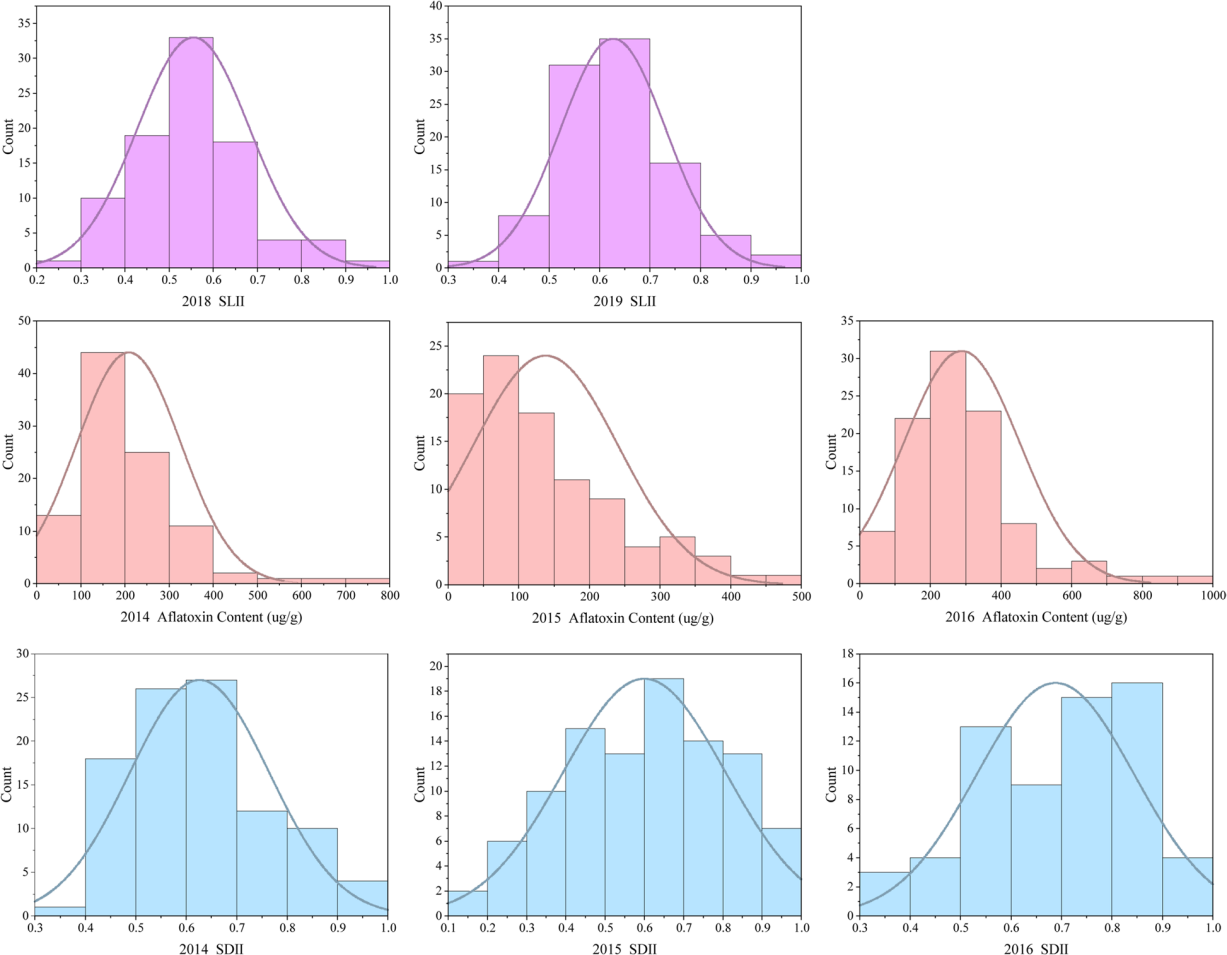
Phenotypic distributions of SLII, SDII and aflatoxin content of Chinese peanut mini-mini core accessions in multiple environments. SLII shell infection index, SDII seed infection index, ENV environment

The 99 peanut accessions of the Chinese mini-mini core were grown and harvested in Wuhan, China in 2014–2016 and 2018–2019. The investigation of shell infection resistance against *A. flavus* of the mini-mini core was implemented in 2018 and 2019 environments, while those for the seed infection resistance and resistance to toxin production were implemented in 2014, 2015 and 2016 environments. For all the above three resistances, artificial inoculation under laboratory conditions with *A. flavus* suspension was performed right after drying of peanut pods. The results showed that the 99 peanut accessions possessed a wide range of variation in the three resistances to *A. flavus* (Table 1). The shell infection index (SLII) ranged from 0.30 to 0.98 across the two years, with the average values being 0.56 and 0.63 in 2018 and 2019, respectively. The seed infection index (SDII) ranged from 0.11 to 0.99 with the average values being 0.63, 0.60 and 0.71 in 2014, 2015 and 2016, respectively. The aflatoxin content in seed ranged from 8.58 to 954.80 μg/g, with the average values being 207.87, 137.93 and 288.96 μg/g in 2014, 2015 and 2016, respectively. The continuous distribution of SLII, SDII and aflatoxin content for the 99 accessions in multiple environments indicated that shell infection resistance, seed infection resistance and aflatoxin production resistance were regulated by multiple genes (Fig. 1).

**Table 1:** Phenotypic variations of SLII, SDII and aflatoxin content of Chinese peanut mini-mini core accessions in multiple environments

Traits	Environment	Range	Mean ± SD	CV
SLII	2018	0.30–0.92	0.56 ± 0.13	0.23
	2019	0.36–0.98	0.63 ± 0.10	0.16
DSII	2014	0.39–0.99	0.63 ± 0.14	0.22
	2015	0.11–0.97	0.60 ± 0.21	0.34
	2016	0.31–0.98	0.71 ± 0.17	0.24
Aflatoxin content (μg/g)	2014	26.90–726.04	207.87 ± 114.68	0.55
	2015	8.58–460.63	137.93 ± 102.80	0.75
	2016	33.28–954.80	288.96 ± 163.90	0.57

*SLII* shell infection index, *SDII* seed infection index, *SD* standard deviation, *CV* coefficient of variation

The correlation analysis of three resistance components was performed and there was no close correlation between SLII and SDII, SLII and aflatoxin content. While there was a significant positive correlation between SDII and aflatoxin content (Table S1). The correlation between the three resistances and pod / seed size related traits were also analyzed. SLII, SDII and aflatoxin content were all positively correlated with pod / seed size (Table S2).

Analysis of variance (ANOVA) indicated that genotype, environment and interaction between genotype and environment significantly affected SLII, SDII and aflatoxin content (*p* < 0.001) (Table 2). The broad-sense heritability for SLII, SDII and aflatoxin content were estimated to be 0.683, 0.847 and 0.641, respectively (Table 2), indicating that all of the three traits were affected by environmental factors.

**Table 2:** Variance analysis of Chinese peanut mini-mini core collection

Traits	Source	DF	SS	MS	F	***P*** value	***h***^***2***^
SLII	Environments	1	0.431	0.431	224.587	<0.001	0.683
	Genotype	97	4.201	0.043	22.574	<0.001	
	Environments×Genotype	90	1.679	0.019	9.725	<0.001	
	Error	290	0.556	0.002			
SDII	Environments	2	1.206	0.603	122.503	<0.001	0.847
	Genotype	98	17.794	0.182	36.886	<0.001	
	Environments×Genotype	193	6.018	0.032	6.401	<0.001	
	Error	494	2.432	0.005			
Aflatoxin content (ug/g)	Environments	2	1,832,234.27	916,117.14	118.693	<0.001	0.641
	Genotype	98	3,659,524.15	37,342.08	4.838	<0.001	
	Environments×Genotype	194	3,211,623.74	16,577.96	2.148	<0.001	
	Error	522	4,028,985.81	7718.36			

*DF* degree of freedom, *SS* sum of squares, *MS* mean square, *h2* broad-sense heritability, *SLII* shell infection index, *SDII* seed infection index

### Identification of elite resistant peanut genotypes

The SLII values of 3 accessions, i.e. Zh.h2193, Zh.h5219 and Zh.h5442, were lower than 0.5 and considered to be moderately resistant to shell infection by *A. flavus*. On the contrary, three accessions, i.e. Zh.h1044, Zh.h3901 and Zh.h3975, had SLII values higher than 0.75 and were considered to be highly susceptible to *A. flavus* infection (Table 3). The SDII values of 7 accessions, i.e. Zh.h0610, Zh.h1197, Zh.h1312, Zh.h1452, Zh.h3216, Zh.h4833 and Zh.h4851, were lower than 0.5 and were considered to be moderately resistant to *A. flavus* infection, five accessions including Zh.h2955, Zh.h3429, Zh.h3901, Zh.h4600 and Zh.h4647, had SDII values higher than 0.75 and were considered to be highly susceptible to *A. flavus* infection (Table 4). Five accessions, Zh.h0530, Zh.h0551, Zh.h1312, Zh.h2150 and Zh.h4302, had low aflatoxin content and were considered to be highly resistant to aflatoxin production, while three accessions (Zh.h3231, Zh.h3429 and Zh.h4600) had high aflatoxin content and were considered to be highly susceptible to toxin production (Table 4). The number of seed infection resistance accessions screened was the largest, followed by aflatoxin production resistance and shell infection resistance. Peanut accessions with low SLII mainly existed in *var. vulgaris* of subspecies *fastigiata* and intermediate type, while accessions with high SLII mainly existed in *var. hypogaea* of ssp. *hypogaea*. Accessions with low SDII mainly existed in *var. hypogaea* of ssp. *hypogaea*, followed by *var*. *vulgaris* of ssp. *fastigiata*. Accessions with low aflatoxin content mainly existed in *var. vulgaris* of ssp. *fastigiata* and *var. hirsuta* of ssp. *hypogaea*. Zh.h1312 had lower SDII value and lower aflatoxin content, and its SLII value was also lower than the susceptible control.

**Table 3:** Peanut accessions with shell infection resistance

Group	Accession number	Shell infection index (SLII)			botanical type
		2018	2019	Mean	
Resistant accessions	Zh.h2193	0.30	0.51	0.40	*var. vulgaris*
	Zh.h5219	0.39	0.43	0.41	*Intermediate*
	Zh.h5442	0.37	0.50	0.43	*Intermediate*
Susceptible accessions	Zh.h1044	0.80	0.81	0.81	*var. hypogaea*
	Zh.h3901	0.90	0.92	0.91	*var. hypogaea*
	Zh.h3975	0.89	0.98	0.93	*var. hypogaea*

**Table 4:** Peanut accessions with both seed infection and aflatoxin production resistance

Traits	Group	Accession Number	2014	2015	2016	Mean	botanical type
SDII	Resistant accessions	Zh.h0610	0.47	0.25	0.52	0.41	*var. hirsuta*
		Zh.h1197	0.44	0.41	0.46	0.44	*var. hypogaea*
		Zh.h1312	0.49	0.16	0.33	0.33	*var. hypogaea*
		Zh.h1452	0.44	0.32	0.39	0.38	*var. hypogaea*
		Zh.h3216	0.42	0.11	0.53	0.35	*var. fastigiata*
		Zh.h4833	0.42	0.20	0.53	0.38	*var. vulgaris*
		Zh.h4851	0.47	0.31	0.43	0.40	*var. vulgaris*
	Susceptible accessions	Zh.h2955	0.89	0.88	0.80	0.85	*var. hypogaea*
		Zh.h3429	0.92	0.94	0.91	0.92	*var. fastigiata*
		Zh.h3901	0.99	0.97	0.98	0.98	*var. hypogaea*
		Zh.h4600	0.86	0.93	0.97	0.92	*var. vulgaris*
		Zh.h4647	0.89	0.94	0.78	0.87	*var. vulgaris*
Aflatoxin content (ug/g)	Resistant accessions	Zh.h0530	149.45	29.33	110.74	96.51	*var. hirsuta*
		Zh.h0551	49.03	14.98	39.76	34.59	*var. hirsuta*
		Zh.h1312	101.83	12.02	75.35	63.07	*var. hypogaea*
		Zh.h2150	52.65	33.60	43.38	43.21	*var. vulgaris*
		Zh.h4302	76.83	112.43	72.30	87.19	*var. vulgaris*
	Susceptible accessions	Zh.h3231	266.70	369.72	647.44	427.95	*var. fastigiata*
		Zh.h3429	354.30	346.16	636.41	445.62	*var. fastigiata*
		Zh.h4600	430.15	404.11	198.25	344.17	*var. vulgaris*

*SDII* seed infection index

### Genotyping of the peanut mini-mini core

The SNP variation of the Chinese peanut mini-mini core collection was identified based on the combination of two genome assembles of the two diploid progenitors of cultivated peanut [23]. Therefore, the previously generated sequencing datasets were re-analyzed using the published genome sequence of the cultivated peanut cultivar Tifrunner (https://www.peanutbase.org/data/public/Arachis_hypogaea/Tifrunner.gnm2.J5K5/) [25] as reference to identify both SNP and InDel variations.

**Fig. 2:**
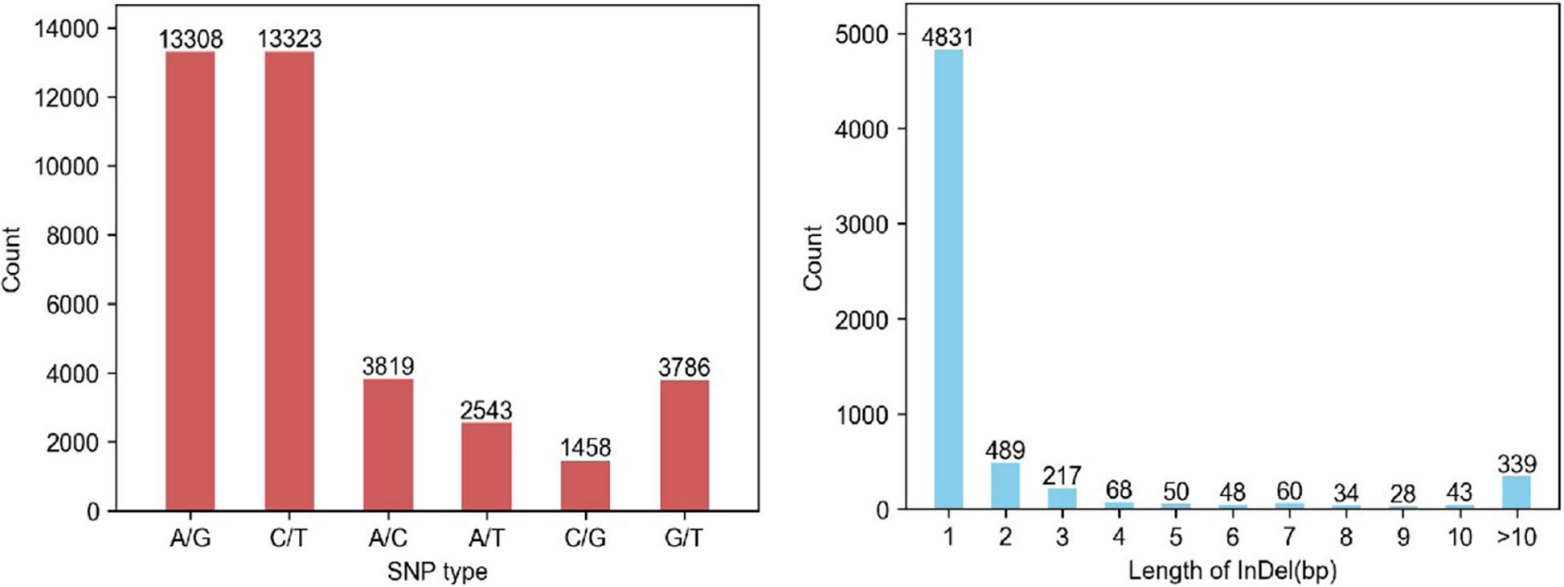
Statistics of variation type

A total of 44,444 polymorphic markers were identified, including 38,237 SNPs and 6207 InDels. Among the SNPs, most of the SNPs were transition types (A/G or C/T) and the transition - transversion ratio (TS/TV) was 2.29 (Fig. 2). The number of SNPs ranged from 922 in chromosome A08 to 3454 in B09, with a mean density of 15.06 SNP/Mb. The number of InDels ranged from 165 in A07 to 552 in B09, with a mean density of 2.45 InDel/Mb (Table 5).

**Table 5:** The number and density of SNP and InDel markers detected across peanut chromosomes

Chr	Length	No. SNPs	SNP density	No. InDels	InDel density
A01	112,420,854	1604	14.27	205	1.82
A02	103,302,290	1406	13.61	203	1.97
A03	143,109,472	2120	14.81	332	2.32
A04	128,801,742	2109	16.37	285	2.21
A05	116,542,366	1853	15.90	248	2.13
A06	118,975,115	1930	16.22	293	2.46
A07	81,752,458	1091	13.35	165	2.02
A08	51,529,986	922	17.89	172	3.34
A09	120,499,698	1714	14.22	223	1.85
A10	117,076,737	1403	11.98	199	1.70
B01	149,287,806	2056	13.77	388	2.60
B02	120,530,088	1709	14.18	286	2.37
B03	146,301,462	1956	13.37	397	2.71
B04	143,237,272	2188	15.28	366	2.56
B05	160,028,458	2319	14.49	364	2.27
B06	151,242,074	2028	13.41	382	2.53
B07	134,191,082	2205	16.43	389	2.90
B08	135,027,066	2494	18.47	436	3.23
B09	159,361,216	3454	21.67	552	3.46
B10	145,034,356	1676	11.56	322	2.22
Total	2,538,251,598	38,237	15.06	6207	2.45

### Population structure and relative kinship

**Fig. 3:**
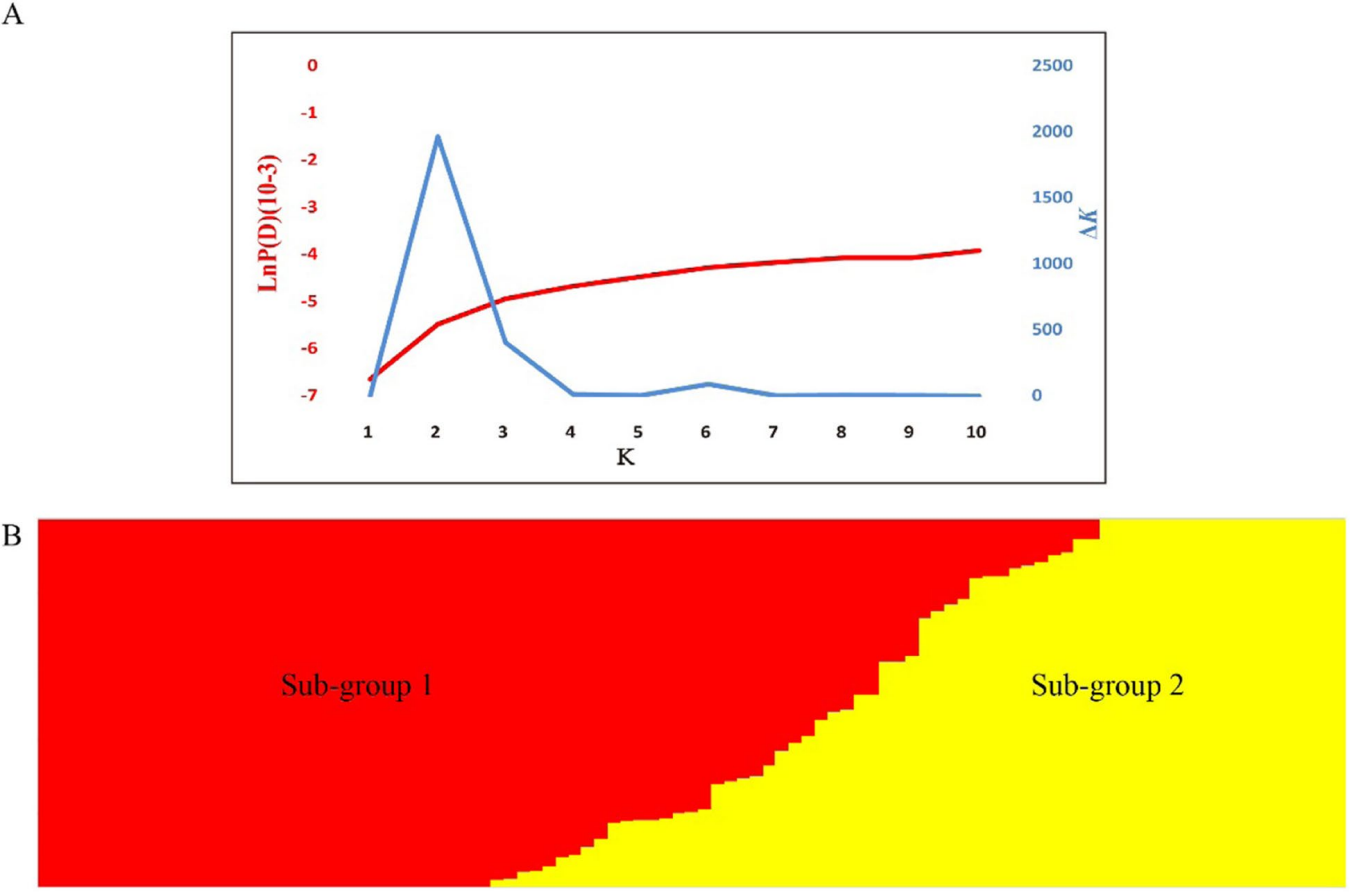
**A** Two different methods for determining the optimal value of *K*. **B** The population structure of 99 peanut accessions when K = 2

The population structure of the 99 peanut accessions in the mini-mini core was assessed using the software STRUCTURE. The significant change of the LnP(D) value was observed, and a sharp peak of delta *K* was observed when K = 2 (Fig. 3A). The population was classified into two subgroups, designated as subgroup 1 and subgroup 2, respectively (Fig. 3B). Subgroup 1 contained 63 accessions, in which 39 accessions (62%) belonged to ssp. *hypogaea*. Thirty-six accessions were classified into subgroup 2, of which 32 accessions (92%) belonged to ssp. *fastigiata* (Table S3). The 99 accessions were also divided into two subgroups using UPGMA (unweighted pair group method using arithmetic average) phylogeny tree based on Nei’s genetic distances, which were basically consistent with the groups estimated by population structure analysis with a few exceptions (Fig. S1). The average relative kinship among the population was 0.088. About 54% kinship estimates in the peanut panel were equal to 0, and more than 60% kinship estimates were below 0.05 (Fig. S2). The results indicated that most lines had no or very weak kinship, which was consistent to the high genetic diversity of the Chinese mini-mini core collection.

### Genome wide association analysis of three resistances

**Fig. 4:**
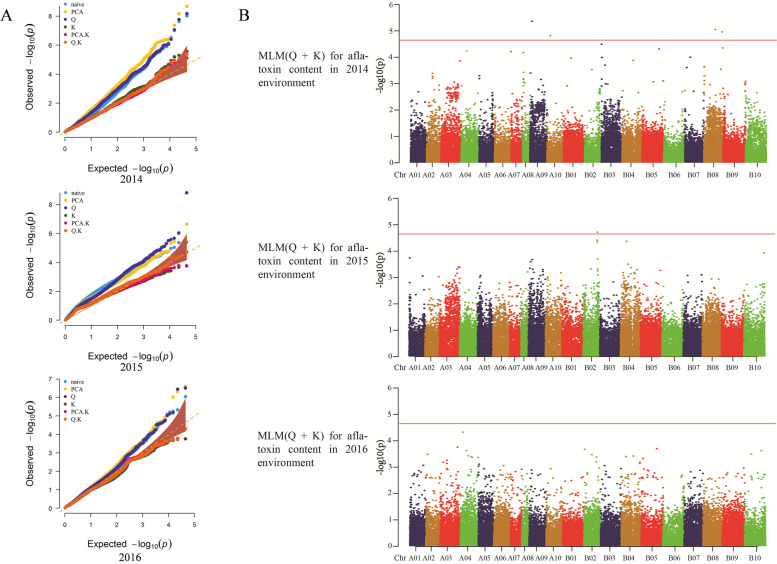
**A** QQ plots for aflatoxin content from 2014 to 2016. **B** Manhattan plots for aflatoxin content from 2014 to 2016

GWAS was performed using TASSEL 5.0 software, and the MLM (Q + K) model, the best among the six tested models according to the results of QQ plots (Fig. 4, Fig. S3, Fig. S4), was selected for GWAS analysis. A total of 6 SNPs and InDels associated with SLII were detected (Fig. S3, Table 6). Only one SLII-associated SNP was detected in 2018, which was located on chromosome A01 with a PVE of 38.64%. Three SLII-associated SNPs and two InDels were detected in 2019 with the PVEs ranging from 24.80 to 33.74% and located on chromosomes B02 (3 SNPs and 1 InDel), B10 (1 InDel) respectively. SNP21070 on chromosome B02 had the largest effect (PVE = 33.74%). Five SNPs and InDels associated with SDII were detected (Fig. S4, Table 6), three SDII-associated SNPs and one InDel were detected in 2014 with their PVEs ranging from 25.82 to 27.62% and located on chromosomes B02 (2 SNPs), B08 (1 InDel) and B10 (1 SNP) respectively, one SDII-associated SNP was detected in 2016, which was located on chromosome B10 with a PVE of 23.33%. InDel36369 on chromosome B08 had the largest effect (PVE = 27.62%). Five SNPs and InDels associated with aflatoxin content were detected (Fig. 4, Table 6), two aflatoxin content associated SNPs and two InDels were detected in 2014 with the PVEs ranging from 30.41 to 36.76% and located on chromosomes A09 (1 InDel), A10 (1 InDel) and B08 (2 SNPs), respectively, one aflatoxin content associated SNP was detected in 2015, which was located on chromosome B02 with a PVE of 34.91%. InDel17247 on chromosome A10 had the largest effect (PVE = 36.76%). By comparative analysis, Zh.h1312 possessed all the resistance alleles of associated loci for SDII and aflatoxin content, which might explain why this accession showed seed infection resistance and aflatoxin production resistance (Table 7). The distribution of SNPs and Indels was shown in Table S4, SNP21070 and SNP42459 were intronic variant of the gene *Arahy.1M3GV3* and *Arahy.36ACQJ*, respectively. SNP22555 was upstream variant of the gene *Arahy.C3WWRZ*, the other associated loci were in intergenic region.

**Table 6:** Significant markers associated with SLII, SDII and aflatoxin content

Traits	Environment	Marker	Chromosome	Position	*P*-Value	PVE
SLII	2018	SNP00688	1	40,368,467	2.07E-05	38.64%
	2019	InDel21071	12	9,357,931	6.92E-06	24.80%
	2019	SNP21070	12	9,277,537	1.13E-05	33.74%
	2019	SNP21188	12	14,933,055	2.13E-05	31.72%
	2019	SNP21021	12	6,844,062	2.22E-05	27.69%
	2019	InDel43846	20	112,260,595	2.23E-05	26.99%
SDII	2014	SNP22555	12	100,237,479	3.60E-06	27.28%
	2014	SNP22420	12	93,852,971	2.14E-05	26.47%
	2014	InDel36369	18	38,548,488	9.42E-06	27.62%
	2014	SNP42459	20	593,672	1.71E-05	25.82%
	2016	SNP43163	20	53,492,328	2.25E-05	23.33%
Aflatoxin content (ug/g)	2014	InDel15210	9	14,197,913	4.35E-06	30.94%
	2014	InDel17247	10	23,667,814	1.52E-05	36.76%
	2014	SNP37276	18	80,753,727	8.90E-06	30.41%
	2014	SNP38307	18	129,415,431	1.09E-05	33.63%
	2015	SNP22577	12	101,526,304	1.90E-05	34.91%

*SLII* shell infection index, *SDII* seed infection index

**Table 7:** Distribution of putative resistant alleles in Zh.h1312 in three resistances

Putative resistant allele	Zh.h1312	Susceptible check
Marker22555	+	–
Marker22420	+	–
Marker36369	+	–
Marker42459	+	–
Marker43163	+	–
SDII	0.33	0.98^a^
Marker15210	+	–
Marker17247	+	–
Marker37276	+	–
Marker38307	+	–
Marker22577	+	–
Aflatoxin content (ug/g)	63.07	412.29^b^

*SDII* seed infection index, a susceptible check Zh.h3901, b susceptible check Zh.h3429

### Linkage disequilibrium and candidate genes for resistance

**Fig. 5:**
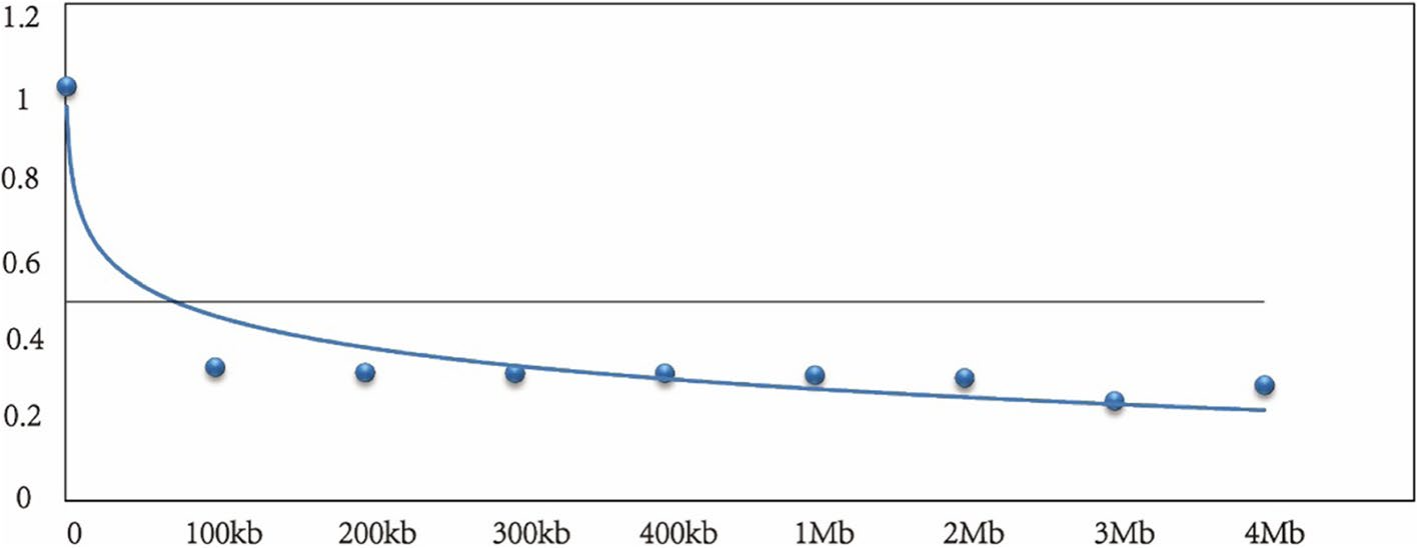
Linkage disequilibrium (LD) decay for the Chinese peanut mini-mini core accessions

Since *r*^*2*^ can reflect the linkage disequilibrium between different loci, *r*^*2*^ was used to calculate the LD decay in this study. The estimated value at 95% of the unlinked *r*^*2*^ was 0.509, so *r*^*2*^ = 0.509 was used as the threshold value for LD decay. The LD decay of the whole genome was calculated to be 80 kb (Fig. 5). Therefore, the candidate genes of SLII, SDII and aflatoxin content associated SNPs/InDels were retrieved within the 80Kb flanking regions (40Kb on upstream and downstream) from the annotation information of the reference genome. A total of 21 SLII associated candidate genes were found for 4 SNPs and 1 InDel (Table S5). *Arahy.J7VJ5I* and *Arahy.7ML2J7* are located at the downstream of SNP21021 and upstream of InDel21071, respectively, and they code MYB transcription factor. The MYB transcription factor was involved in the biosynthesis of terpenoids. In addition, the genes, *Arahy.12GONV* and *Arahy.FTX6XU*, for the synthesis of hydroquinone glycosyltransferase were identified in the candidate interval of InDel21071. Eighteen SDII associated candidate genes were found for 2 SNPs and 2 InDels and fifteen aflatoxin content associated candidate genes were found for 2 SNPs and 2 InDels. *Arahy.R1ATPI* and *Arahy.1ZVJ53* are located at the downstream of SNP22577 which is related to effector receptor (NLR) and could regulate plant disease resistance.

## Discussion

### Variation of resistance to aflatoxin in peanut

As one key factor affecting peanut food safety, aflatoxin contamination seriously restricts the development of peanut industry and may cause waste of food resource. Reducing the risk of aflatoxin contamination is one of the key tasks of peanut research community worldwide.

Many studies have summarized the differentiation of resistance to *A. flavus* in peanut. Khan et al. observed broad range of phenotypic variations among RILs against *A. flavus* infection [19]. Jiang et al. [26] reported continuous distributions with transgressive segregation among RILs in seed infection resistance. Yu et al. [18] also observed transgressive segregation and continuous distribution in RIL population for both seed infection resistance and toxin production resistance. But no systematic evaluation for all the three resistances and their relationship has been carried out up to date. The findings in this study, based on the vast genetic diversity of mini-mini core selected from a basic collection of more than 6500 accessions, showed that there was great differentiation among the three resistances against aflatoxin.

Analysis of variance revealed that SLII, SDII and aflatoxin content were significantly affected by genotype, environment and interaction between genotype and environment (*p* < 0.001) (Table 2). The result of broad-sense heritability also indicated that SLII, SDII and aflatoxin content were partly controlled by genetic factors and affected by environmental factors. The moisture and heat stress, damage of pods by insect pests and nematodes, and other injury during pod development would all facilitate preharvest seed infection. Arunyanark et al. [27] found that higher levels of *A. flavus* inoculum load in the soil under drought conditions resulted in increased kernel colonization and subsequent aflatoxin contamination. As SLII, SDII and aflatoxin content are traits highly affected by environments, the phenotypic values of the same trait varied among different environments in this study. The phenotypic values of the same trait in different environments were significantly correlated (Table S1), which indicated that the phenotypic identification method was effective. For the correlation between different resistances, Mehan et al. [28] reported that there were poor correlation between *in vitro* seed colonization by *A. flavus* (IVSCAF) resistance and post-harvest aflatoxin production. Xue et al. [29] reported that there were no absolute relationships of aflatoxin production resistance with IVSCAF, field resistance to seed colonization by *A. flavus* (FSCAF), or PAC resistance. In this study, the seed infection resistance was significantly positively correlated with toxin production resistance, while the shell infection resistance was not correlated with other two resistances. The correlation among the three resistances revealed the potential of synergistic improvement of resistance.

### Novel germplasmwith shell infection resistance

Through the evaluation of shell infection resistance, seed infection resistance and aflatoxin production resistance of the Chinese peanut mini-mini core accessions across multiple environments, a total of 14 resistant accessions were identified, among them, three accessions were resistant to shell infection, seven were resistant to seed infection, and five were resistant to aflatoxin production. These accessions could be used to further investigate the mechanism and molecular basis of resistance to *A. flavus*. Zh.h1312 was resistant to both seed infection and toxin production, meanwhile the shell infection resistance of Zh.h1312 was also much better than the susceptible control, showing being an ideal parent in breeding. As for the screening of resistant germplasm, five accessions were screened for aflatoxin production resistance in this study are not exactly the same as Yu’s [23] study due to the different methods used in screening. More research would be conducted to reveal the role of these resistant accessions in reducing post-harvest infection under field conditions. Mehan et al. [30] reported J11 had a significantly lower aflatoxin content than susceptible control tested while Anderson et al. [31] observed that J11 had no resistance to aflatoxin contamination in the field studies. There could be many reasons for this contradictory result, such as different locations and soil types, different inoculation times or methods.

### GWAS and genes associated with three resistance components

Quantitative trait loci (QTL) or genome-wide association analysis can be used to quickly identify molecular markers and genes associated with traits, which is a very effective method to map candidate genes. Important progress has been made in mapping complex quantitative traits of peanut by GWAS [32–34]. However, a few GWAS or QTL research has been made for seed infection resistance and toxin production resistance, no research has been conducted to identify QTLs or their associated genes related to shell infection resistance in peanut. This is the first GWAS study to identify associated molecular markers for shell infection resistance, seed infection resistance and aflatoxin production resistance. Population structure and relative kinship were calculated to control false-positive results in GWAS. A total of six SNPs/InDels distributed on three chromosomes were detected to be associated with shell infection index in 2018 and 2019 environments, five SNPs/InDels distributed on four chromosomes were detected to be associated with seed infection index, and five SNPs/InDels distributed on four chromosomes were detected to be associated with aflatoxin content in 2014, 2015 and 2016 environments. The three resistances associated SNPs/InDels were located at different locations, suggesting that the three resistances might be controlled by different genes. Upadhyaya et al. [24] indicated that the resistance to preharvest seed infection, *in vitro* seed colonization (IVSC) and aflatoxin production are inherited independently. The levels of resistance could be improved further by pyramiding different resistance genes.

Pod shell is the first physical defense mechanism against fungal infection to peanut kernels. The shell infection resistance is attributed to the structure and the content of various components such as the content of lignin, cellulose, monosaccharide and disaccharide. Among the candidate genes identified, *Arahy.J7VJ5I* and *Arahy.7ML2J7* encode MYB transcription factor. Kishi-Kaboshi et al. [35] found that the MYB transcription factors MYB30, MYB55 and MYB110 could lead to the accumulation of ferulic acid by activate the cinnamate/monolignol synthesis genes to confer resistance to both fungal and bacterial pathogens in rice. In addition, MYB transcription factors (TFs) have important roles in regulating lignin biosynthesis. He et al. [36] found that OsMYB30 mediate the resistance to brown planthopper by directly up-regulated the expression of *OsPAL6* and *OsPAL8* genes to increase the content of salicylic acid and lignin in rice. Zhang [37] reported a MYB TF encoding gene, *EjODO1*, regulated lignin biosynthesis in the fruit of loquat (*Eriobotrya japonica*). Koshiba [38] found the heterologous expression of AtMYB61 in rice increased lignin content mainly by enriching syringyl units as well as p-coumarate and tricin moieties in the lignin polymers. Two genes *Arahy.12GONV* and *Arahy.FTX6XU* for the synthesis of glycosyltransferase were identified in the candidate interval labeled by InDel21071. The function of glycosyltransferases in some plants has been confirmed to promote lignification by glycosylating secondary metabolites in plants. Glycosylation that transports lignin precursors from intracellular to extracellular membrane is very important for lignin synthesis. Dong et al. [39] mapped a QTL *GSA1* that regulates both grain shape and stress resistance in rice by map-based cloning. *GSA1* can utilize lignin monomers, such as conibenol, p-coumarol and myrosinol, as glycogen transfer receptors to regulate lignin content. Therefore, the resistance of peanut pod shell to fungi infection might be related to lignin amount.

Two genes, *Arahy.R1ATPI* and *Arahy.1ZVJ53,* are located at the downstream of SNP22577 which was related to effector receptor (NLR). Plants could sense the invasion of pathogens through specific recognition of pathogenic effector proteins, and then activate a rapid and accurate immune response. As a member of phosphorylation pathway, Arabidopsis crck3 (calmodulin binding receptor like cytoplasmic kinase 3) is recognized by NLR protein summa (suppressor of mkk1 mkk2 2) after phosphorylation, which further promotes the occurrence of immune response [40]. In the process of seed resistance to toxin production, peanut seed may perceive the invasion of *A. flavus* through the specific recognition of *A. flavus* effector protein, and then activate the immune response.

## Conclusions

There was a wide variation in the three resistances against *A. flavus* among the Chinese peanut mini-mini core collection, and 14 resistant accessions were discovered. Among the mini-mini core, the ratio of accessions of seed infection resistance was the highest, followed by toxin production resistance, while that of shell infection resistance was the lowest. For the distribution of resistant accessions among subspecies and botanical types, all the three resistances were found in *var. vulgaris* of ssp. *fastigiata*. In terms of correlation of the three resistances, there was a positive correlation between seed infection resistance and toxin production resistance, but there was no close correlation between these two resistances with the shell infection resistance. For the relationship between *A. flavus* resistance and other important characters, there was a significant negative correlation between the three resistances and pod and/or seed size. Through comprehensive evaluation, a special accession, Zh.h1312 with seed infection resistance and aflatoxin production resistance was identified as a novel resistance source for breeding. Meanwhile, this accession was also revealed to possess all the resistance alleles of associated loci for seed infection index and aflatoxin content. The revealed differentiation in resistance to *A. flavus*, identified accessions and the diagnostic markers discovered in this study would provide important basis for intensifying the aflatoxin resistance improvement especially for pyramiding the three resistance components in peanut.

## Methods

### Peanut germplasm materials and *A. flavus* strain

A total of 99 accessions of the Chinese mini-mini core collection were used in the present study. The above peanut accessions were normally maintained in the genebank at the Oil Crops Research Institute of Chinese Academy of Agricultural Sciences (OCRI-CAAS), China. The materials were planted in the same experimental field as in the previous study [23] in Wuhan, China in two growing seasons (2018–2019). After harvest, all peanut pods were dried to 5–8% moisture content. Healthy, mature and undamaged pods were selected for further experiment.

The *A. flavus* strain AF2202 with strong capacity of seed invasion, colonization and consequent aflatoxin production, maintained in 20% glycerol (−80 °C) at OCRI-CAAS, was used for artificial inoculation and screening for shell infection resistance.

### Phenotypic evaluation of shell infection resistance against *A. flavus*

The method established by Qiu et al. [16] for screening shell infection resistance was used in the present study. AF2202 was cultured on fresh potato dextrose agar medium at 30 °C for 7 days, and conidia were then collected and suspended in sterile water containing 0.05% Tween-80. The concentration of conidia was determined at ~2 × 10^6^ conidia/ml using haemocytometer and used to inoculate peanut pods.

Before inoculation, 15 mature, healthy and undamaged peanut pods of each accession were surface-sterilized by immersion in 1% sodium hypochlorite commercial solution for 13 min, and washed with sterile distilled water for three times. Then 15 peanut pods were placed in a 12 cm Petri dish, and then 1.0 ml conidial suspension of *A. flavus* was added into the dish. The conidia of *A. flavus* were evenly distributed on the pod surface by gently oscillating the Petri dish. Finally, the dish was incubated at 30 °C in dark for 7 days. The infection degree of each pod was determined according to the coverage of spore and thick spores on shell surface. The infection degree of each pod were recorded in a 0–8 scale as shown in Table 8 and Fig. S5. The shell infection index (SLII) of each accession was calculated with the formula $\mathrm{SLII}=\frac{\sum_{i=0}^8i\times Ni}{8\times N}$, where *N* indicate the number of pods in total, i indicate the scale of infection degree of each pod, and *Ni* indicate the number of pods classified as scale i. The peanut accession with SLII less than 0.25 was defined as highly resistant, 0.25–0.50 as moderately resistant, 0.50 to 0.75 as moderately susceptible, and 0.75 to 1 as highly susceptible.

**Table 8:** Classification of scales of infection degree of peanut pod shell by *A. flavus*

Scale	0	1	2	3	4	5	6	7	8
The percentage of shell surface covered by spores (%)	0	0–20	20–40	40–60	60–80	80–100	100	100	100
The percentage of shell surface covered by thick spores (%)	0	0	0–5	5–10	10–30	30–50	50–70	70–90	90–100

The methods for screening the seed infection resistance and determining the aflatoxin content were as described by Yu et al. [18]. Seed infection values were obtained from laboratory investigation from 2014 to 2016, aflatoxin content values were obtained from Yu et al. [23] while the method of screening resistant materials is different from his. The grouping criteria of seed infection resistance were consistent with the shell infection resistance. For the screening of resistant accessions, we sorted the phenotypic values of each environment, and then selected the top 10% materials. The materials repeatedly screened in two or more environments were the resistant materials screened. Susceptible accessions were screened in the same way.

### Statistical analysis

Descriptive statistical analyses and analysis of variance (ANOVA) test of the phenotypic data were performed with SPSS Statistics 25.0 statistical software.

### Genotyping and genetic diversity analysis

The RAD-seq reads of the 99 peanut accessions of the Chinese mini-mini core were generated by Yu et al. [23]. In this study, the tetraploid reference genome was be used for genotype calling on the RAD-seq data. These clean reads were mapped to the peanut reference genome (https://www.peanutbase.org/data/public/Arachis_hypogaea/Tifrunner.gnm2.J5K5/) using BWA software. The SAMTools software was used to convert the SAM format files generated by BWA into BAM format. The HaplotypeCaller module in GATK software package was used to generate gvcf files for each accession, and then the GenotypeGVCFs module was used to identify SNPs and InDels of all the 99 accessions. SNPs and Indels with MAF less than 5%, or with alleles more than 2, or missing rate higher than 0.5 were filtered out.

### Population structure and relative kinship

The STRUCTURE v2.2 software [41] was used to estimate population structure (Q-matrix). The optimal number of subgroups K was set as 1–10, and the number of iterations was set as 5. The other default parameters of the software were unchanged. The best K value was calculated to determine the number of subgroups according to Evanno and Regnaut [42]. The phylogenetic tree was generated by the UPGMA clustering method using TASSEL 5.0 softwarebased on the genotype of the 99 accessions. The relative kinship estimation matrix was generated using the EMMAX software [43], and all negative kinship values were set to zero [44].

### GWAS for shell infection index

The general linear model (GLM) and mixed linear model (MLM) were performed in TASSEL 5.0 software [45] to conduct genome-wide association analysis (GWAS) with population structure (Q value) and the kinship matrix (K value) as covariates. Six statistical models were used for association analysis, including naive, Q and PCA models in GLM as well as K, Q + K and PCA + K models in MLM. According to the -log_10_(*P*) observation value and expected value of each SNP, the R software was used to draw quantile-quantile scatter plot (QQ plot). The best model was determined by comparing QQ plot, and the GWAS of SLII, SDII and aflatoxin content were carried out under the optimal model. The total number of SNPs/InDels used in GWAS were 44,444, and *P*-value threshold was calculated by using the Bonferroni method (p ≤ 1/44,444 = 2.25 × 10^−5^, −log_10_P ≥ 4.65). SNPs/InDels with *P* value less than the threshold were significant association sites for the three resistances. Candidate genes were identified with the LD intervals of significant association sites.

### Linkage disequilibrium

LD was estimated with the correlation coefficient *r*^*2*^ between all SNP and InDel makers using TASSEL 5.0 software [45]. SNPs and InDel markers on the same chromosome were considered as linked markers, while markers from different chromosomes as unlinked markers.

According to the suggestion of Breseghello and Sorrells [46], the *r*^*2*^ of unlinked markers were considered as a population-specific background linkage disequilibrium. After eliminating the background linkage disequilibrium, the remaining LD was considered to be caused by linkage. In this study, *r*^*2*^ value greater than 95% of unlinked *r*^*2*^ was used as the baseline, which is the threshold of LD decay. Linked *r*^*2*^ values were classified into a series interval of 0–100 kb, 100–200 kb, 200–300 kb, 300–400 kb, 400 kb-1 Mb, 1–2 Mb, 2–3 Mb, and 3–4 Mb based on variation distances, then the average *r*^*2*^ value for each interval were calculated. The *r*^*2*^ value for 0 kb interval was assumed to be 1 as previously described [47]. The best fitting curve was obtained to describe the LD decay by nonlinear regression model.

### Availability of data and materials

Sequencing data are deposited in Sequence Read Archive of NCBI under the BioProject accession number PRJNA797207, the data will be released on 2023-02-01, before which information can be obtained through the link below (https://dataview.ncbi.nlm.nih.gov/object/PRJNA797207). All data generated or analyzed during this study are included in this article (and its supplementary information files) or are available from the corresponding author on reasonable request.

## Supplementary Information

**Additional file 1: Figure S1.** Dendrogram of the peanut panel based on genotypic data. **Figure S2.** Frequency distribution of relative kinship coefficient in 99 peanut accessions. **Figure S3.** (A) QQ plots for shell infection index (SLII) in 2018 and 2019. (B) Manhattan plots for SLII in 2018 and 2019. **Figure S4.** (A) QQ plots for seed infection index (SDII) from 2014 to 2016. (B) Manhattan plots for SDII from 2014 to 2016. **Figure S5.** 0-8 scale for resistance screening of peanut pod.

**Additional file 2: Table S1.** Correlation analysis of three resistances. **Table S2.** Correlation analysis between three resistances and other traits. **Table S3.** The information of the 99 accessions which are derived into two sub-groups. **Table S4.** The distribution of SNPs and Indels. **Table S5.** The information of candidate genes.

## Abbreviations

SLII: Shell infection index
SDII: Seed infection index
*A. flavus*: *Aspergillus flavus*
GWAS: Genome-wide association study
ICRISAT: International Crops Research Institute for the Semi-Arid Tropics
LD: Linkage disequilibrium
SNP: Single nucleotide polymorphism
IVSCAF: *In vitro* seed colonization by *A. flavus*
FSCAF: Field resistance to seed colonization by *A. flavus*
GLM: General linear model
MLM: Mixed linear model
