# Supplementary material for: Comprehensive evaluation of Chinese peanut mini-mini core collection and QTL mapping for aflatoxin resistance
Source: BMC Plant Biol. 2022 Apr 21;22:207. doi: 10.1186/s12870-022-03582-0 (PMC9027753; doi:10.1186/s12870-022-03582-0)
Supplement: Supplementary file 1 — Additional file 1: Figure S1. Dendrogram of the peanut panel based on genotypic data. Figure S2. Frequency distribution of relative kinship coefficient in 99 peanut accessions. Figure S3. (A) QQ plots for shell infection index (SLII) in 2018 and 2019. (B) Manhattan plots for SLII in 2018 and 2019. Figure S4. (A) QQ plots for seed infection index (SDII) from 2014 to 2016. (B) Manhattan plots for SDII from 2014 to 2016. Figure S5. 0-8 scale for resistance screening of peanut pod. [file 12870_2022_3582_MOESM1_ESM.docx]

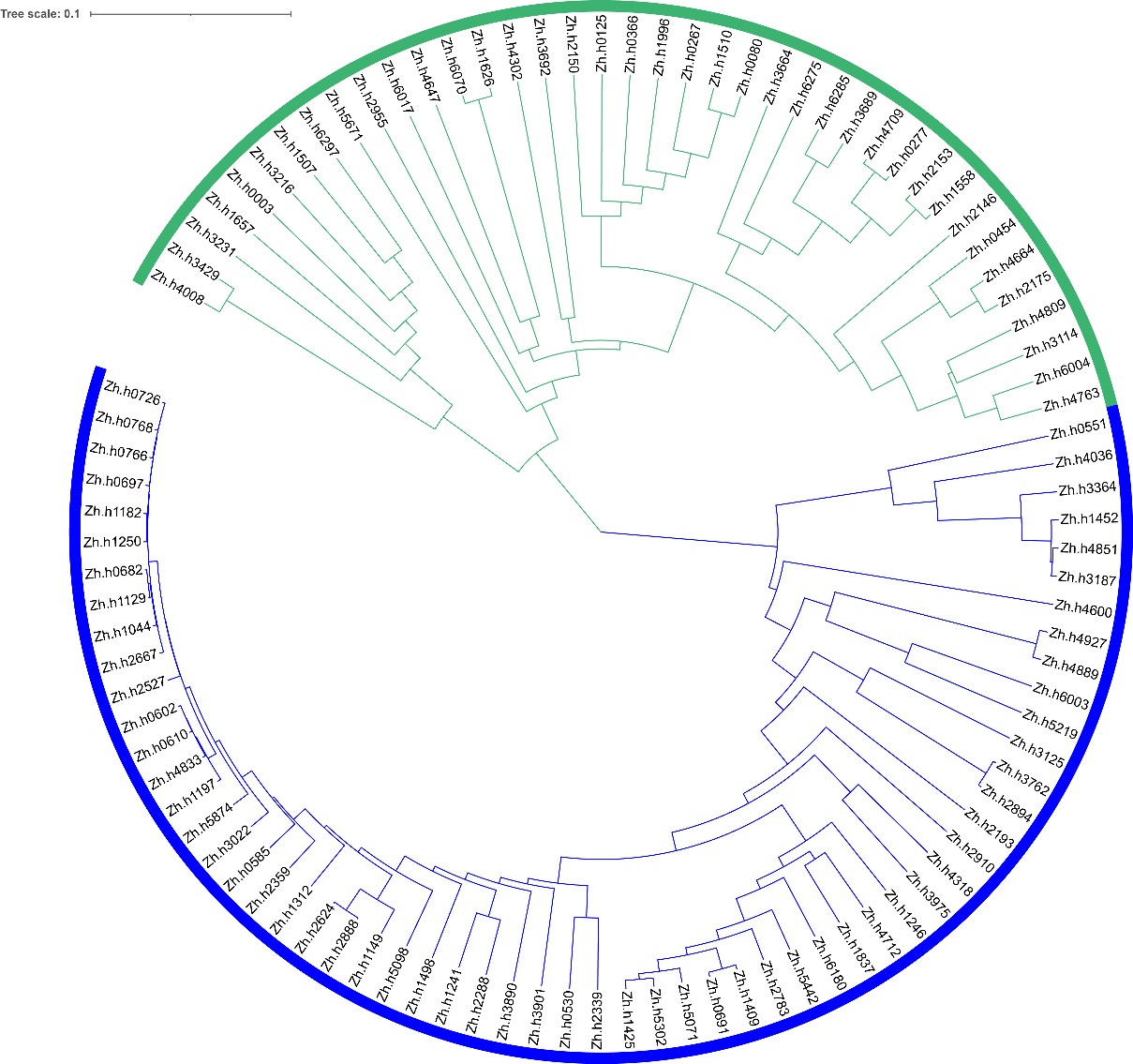


**Figure S1** Dendrogram of the peanut panel based on genotypic data

**Figure S2** Frequency distribution of relative kinship coefficient in 99 peanut accessions. Only kinship values ranged from 0 to 0.5 were shown.


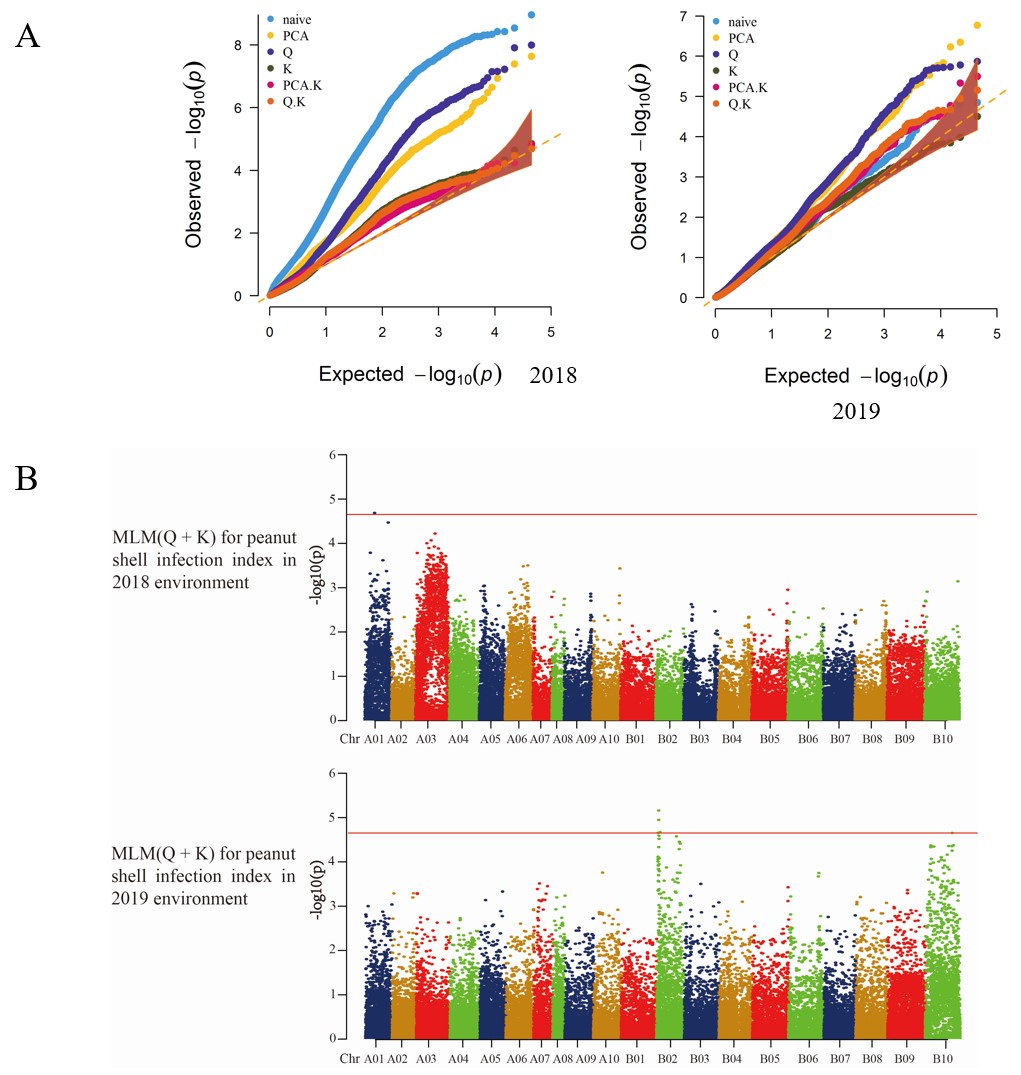


**Figure S3** (A) QQ plots for shell infection index (SLII) in 2018 and 2019. (B) Manhattan plots for SLII in 2018 and 2019.


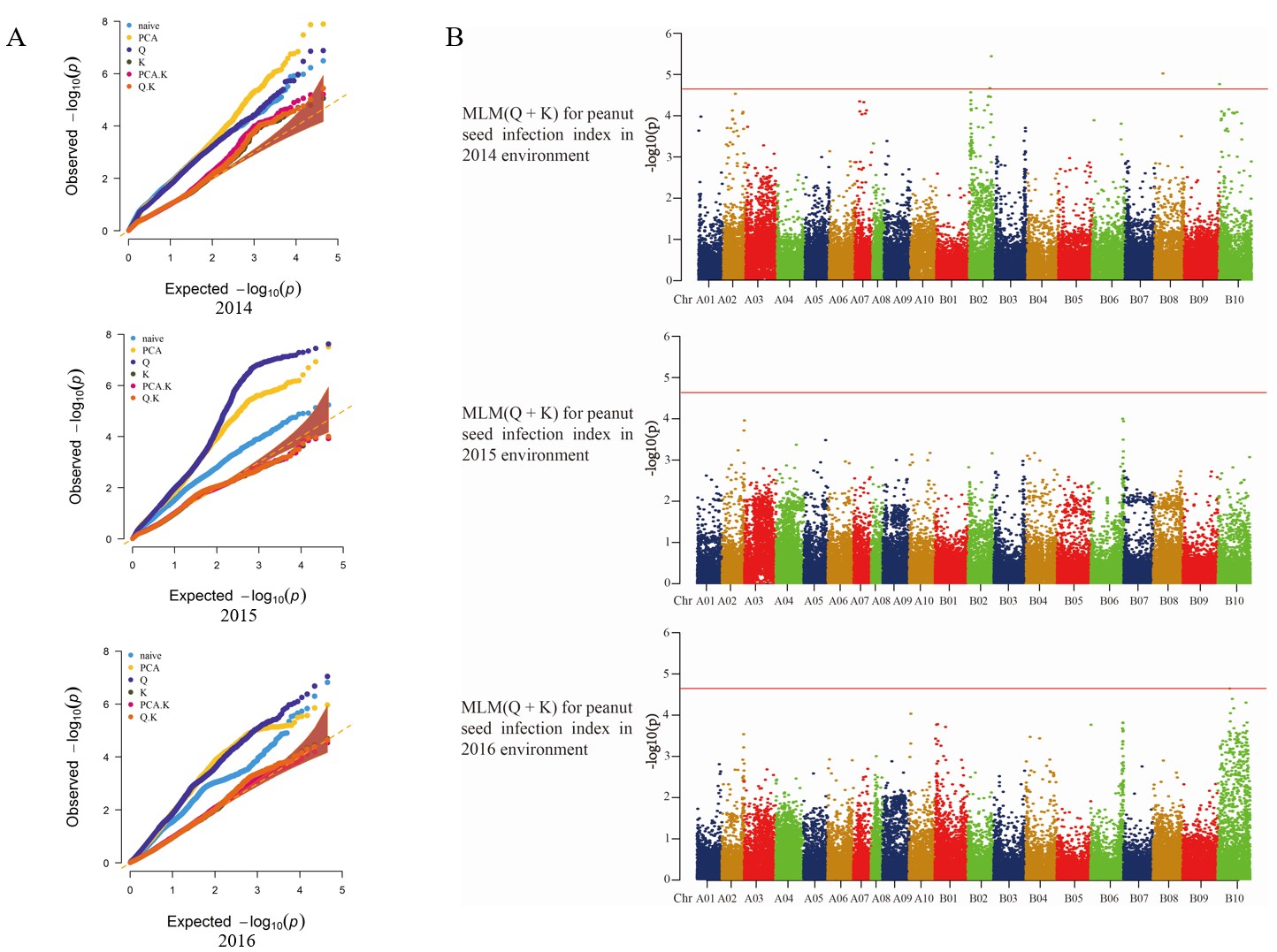


**Figure S4** (A) QQ plots for seed infection index (SDII) from 2014 to 2016. (B) Manhattan plots for SDII from 2014 to 2016.

**Figure S5** 0-8 scale for resistance screening of peanut pod
